# HLA-DM catalytically enhances peptide dissociation by sensing peptide–MHC class II interactions throughout the peptide-binding cleft

**DOI:** 10.1074/jbc.RA119.010645

**Published:** 2020-01-22

**Authors:** Eduardo Reyes-Vargas, Adam P. Barker, Zemin Zhou, Xiao He, Peter E. Jensen

**Affiliations:** ‡ Department of Pathology, University of Utah School of Medicine, Salt Lake City, Utah 84112; § Department of Pathology, ARUP Institute for Clinical and Experimental Pathology, ARUP Laboratories, Salt Lake City, Utah 84108

**Keywords:** antigen presentation, antigen processing, major histocompatibility complex (MHC), peptide interaction, kinetics, fluorescence anisotropy, catalysis, Michaelis-Menten, immunogenicity, immunology

## Abstract

Human leukocyte antigen-DM (HLA-DM) is an integral component of the major histocompatibility complex class II (MHCII) antigen-processing and -presentation pathway. HLA-DM shapes the immune system by differentially catalyzing peptide exchange on MHCII molecules, thereby editing the peptide-MHCII (pMHCII) repertoire by imposing a bias on the foreign and self-derived peptide cargos that are presented on the cell surface for immune surveillance and tolerance induction by CD4^+^ T cells. To better understand DM selectivity, here we developed a real-time fluorescence anisotropy assay to delineate the pMHCII intrinsic stability, DM-binding affinity, and catalytic turnover, independent kinetic parameters of HLA-DM enzymatic activity. We analyzed prominent pMHCII contacts by differentiating the kinetic parameters in pMHCII homologs, observing that peptide interactions throughout the MHCII-binding cleft influence both the rate of peptide dissociation from the DM-pMHCII catalytic complex and the binding affinity of HLA-DM for a pMHCII. We show that the intrinsic stability of a pMHCII linearly correlates with DM catalytic turnover, but is nonlinearly correlated with its binding affinity. Surprisingly, interactions at the peptides N terminus up to and including MHCII position one (P1) anchor affected the catalytic turnover, suggesting that the active DM-pMHCII catalytic complex operates on pMHCII complexes with full peptide occupancy. Furthermore, interactions at the peptide C terminus modulated DM-binding affinity, suggesting distal communication between peptide interactions with the MHCII and the DM-pMHCII binding interface. Our results imply an intimate linkage between the DM-pMHCII interface and peptide-MHCII interactions throughout the peptide-binding cleft.

## Introduction

CD4^+^ T cells play a central role in adaptive immunity. CD4^+^ T cell antigen receptors recognize proteolytically processed protein fragments (peptides) bound to major histocompatibility complex class-II (MHCII)^3^ proteins on the surface of antigen-presenting cells. The MHCII antigen-processing and -presentation pathway is crucial for immune surveillance as the mechanism that generates the ligands that engage with CD4^+^ T cells (1, 2). Within the pathway, the human leukocyte antigen-DM (HLA-DM) protein enhances peptide loading of MHCII complexes by differentially catalyzing peptide exchange reactions on classical MHCII molecules (3–10). Through this action, HLA-DM markedly impacts the specificity of antigen presentation by continuously proofreading the peptide-MHCII complexes that are en route for presentation to CD4^+^ T cells (8, 9, 11–13).

HLA-DM is structurally homologous to classical MHCII molecules. Despite the similarity, DM is unable to associate with peptide antigens due to the near-complete occlusion of the peptide-binding cleft (14). Classical MHCII molecules associate with peptide cargo through a network of MHCII conserved hydrogen bonds with the peptide main chain and side chain occupancy of several anchor pockets that vary in size and chemical features (Fig. S1, *A* and *B*). Relative peptide occupancy of these pockets within the binding cleft designates peptide anchor residues at positions 1 (P1), 4 (P4), 6 (P6), 7 (P7), and 9 (P9). Stable peptide-MHCII complexes are formed when anchor pockets are occupied with chemically and structurally compatible side chains. These optimal amino acid residues define the peptide-binding motif of the MHCII.

HLA-DM encodes an endogenous signal that directs steady-state localization into late endosomal MHC class II compartments (MIIC) where antigen processing occurs (15, 16). Classical MHCII dimers instead associate with the invariant chain (li) chaperone protein to facilitate transport into the MIIC where resident cathepsins process the invariant chain protein into the class II-associated invariant chain peptide (CLIP), converting all li-MHCII trimers into CLIP-MHCII complexes (17). These complexes are the earliest that DM encounters during the 2–4 h dwell time within the MIIC. To promote effective immune surveillance, DM must first catalyze the removal of CLIP and enhance the loading of antigenic peptides onto an MHCII (4, 5, 18, 19). Throughout the endosomal dwell time, DM continues to catalyze peptide exchange through a mechanism that involves a transient interaction with the pMHCII complex. HLA-DM's enzymatic activity varies with each pMHCII species, such that peptide exchange is markedly enhanced for some pMHCII complexes, whereas others remain relatively unaffected (8, 9, 11, 12, 20, 21). This functional variability imposes a bias on the pMHCII that skews the repertoire of foreign and self-derived peptides that are presented to CD4^+^ T cells for activation or tolerance induction. It is through this effect that HLA-DM is said to edit the pMHCII repertoire that is presented on the cell surface.

Although a large body of evidence demonstrates a central role for HLA-DM in shaping T cell immunity, neither DM's catalytic mechanism nor the pMHCII structural or chemical determinants that govern differential susceptibility to DM editing are well-understood (4, 8–10). Early investigations reported that in the absence of HLA-DM, empty MHCII molecules rapidly transitioned into an unreceptive peptide-binding conformation (22, 23). In the presence of HLA-DM, a highly receptive peptide-binding state was maintained. These studies led to the proposal that DM accelerates peptide loading by stabilizing a peptide-receptive transition state that is maintained until a suitable peptide binder is encountered (24, 25). Recent studies, however, do not support this hypothesis (26). Further results have provided evidence that DM's enzymatic activity is related to the intrinsic stability of the pMHCII, such that less stable complexes are selectively edited (8, 9, 20, 21). These studies support the idea that HLA-DM preferentially dissociates peptides from kinetically unstable pMHCII complexes and forms a peptide vacant DM-MHCII intermediate that resolves upon binding a high-affinity peptide, thereby selecting the most intrinsically stable pMHCII complexes for presentation. This hypothesis has been challenged by studies demonstrating examples of DM editing of stable pMHCII complexes, suggesting the presence of unidentified factors in the susceptibility of pMHCII complexes to the enzymatic activity of HLA-DM (11, 27, 28).

The possibility that DM targets the conserved hydrogen-bond network between MHCII and the peptide main chain has been investigated (Fig. S1*A*) (8, 29–32). Our group and others have since shown that no individual bond within the network is essential for HLA-DM's catalytic activity (33–35). Recent studies have identified a region in the pMHCII near the peptide N terminus that substantially impacts its susceptibility to DM editing (32, 36–38). This finding has advanced the idea that interactions at the peptide N terminus may be the fundamental determinant of HLA-DM's enzymatic activity (27, 39, 40). In a seminal work by Pos *et al.* (41), two co-crystal structures (pH 5.5 and 6.5) were solved for HLA-DM bound to HLA-DR1 covalently tethered to an N-terminal–truncated hemagglutinin (HA) peptide. The structures showed that HLA-DM does not obstruct the peptide-binding cleft and confirmed that the binding interface consists mainly of interactions between residues in the DRα1 and DMα1 domains. Moreover, the structures revealed significant conformational alterations to the MHCII in a region proximal to the peptide N terminus. This landmark study proposed the prevailing model of DM-mediated peptide exchange, whereby binding of HLA-DM to a pMHCII requires the partial dissociation of peptide from the MHCII-binding cleft (34, 41, 42). This model predicates that dissociation of the peptide N terminus is the key determinant of HLA-DM susceptibility. Other studies, however, have suggested that DM susceptibility may not be entirely determined by disruptions at the peptide's N terminus. Rather, DM susceptibility may be governed by the global conformational state of the pMHCII (20, 28, 43, 44).

In the current study, we find that DM-mediated peptide dissociation rates are saturable. This observation allowed us to investigate pMHCII susceptibility to HLA-DM editing by resolving the independent kinetic parameters in the enzymatic activity of DM-mediated peptide exchange reactions. We developed a fluorescence anisotropy assay that measured the real-time dissociation of peptides from MHCII molecules and derived a Michaelis-Menten kinetic model that equates the peptide's observed dissociation rate (*k*_obs_) with pMHCII intrinsic stability (*k*_in_), DM binding affinity (*K_m_*), and catalytic turnover (*k*_cat_). Analyzing multiple pMHCII complexes, we find that the intrinsic stability of a pMHCII is linearly correlated with DM catalytic turnover and nonlinearly correlated with DM-binding affinity. We evaluated the impact of prominent pMHCII structural and chemical features on the catalytic mechanism and discovered that interactions throughout the peptide-binding cleft contribute to the binding affinity of DM and to the rate of peptide dissociation from the catalytic complex. We provide evidence that dissociation of the peptide N terminus is not a necessary precondition for binding of HLA-DM to pMHCII complexes.

## Results

### HLA-DM–catalyzed peptide dissociation rates are saturable, enabling the independent kinetic parameters in the catalytic mechanism to be determined

**Figure 1.**
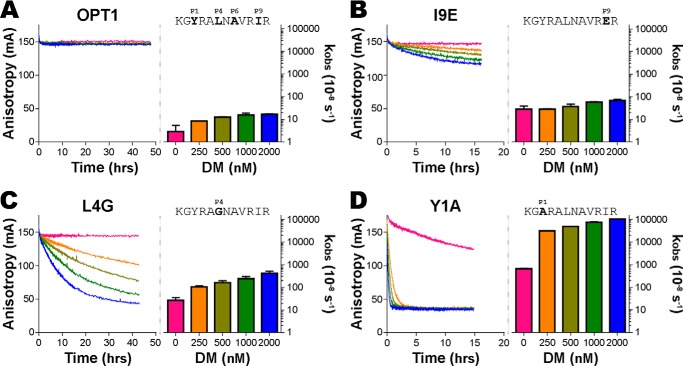
**HLA-DM–mediated peptide dissociation of an optimal binder is enhanced by single amino acid substitutions.** Fluorescence anisotropy (*left*) is shown for the real-time dissociation of fluorescence-labeled peptides bound to HLA-DR1 in the presence of titrated concentrations of HLA-DM. Anisotropic dissociation curves were modeled by a single-phase exponential decay equation to determine the observed peptide dissociation rate (*k*_obs_) for each concentration of DM (*right*). *A,* the OPT1 peptide-complex contains optimal binding residues for anchors at position 1 (Tyr), position 4 (Leu), position 6 (Ala), position 7 (Val), and position 9 (Ile), and is minimally enhanced by concentrations of up to 2 μm HLA-DM. *B,* peptide dissociation is enhanced by 1 order of magnitude by an isoleucine to glutamic acid substitution at anchor position 9 (I9E). *C,* dissociation is enhanced by 2 orders of magnitude by a leucine to glycine substitution at anchor position 4 (L4G). *D,* peptide dissociation is enhanced by 4 orders of magnitude by a tyrosine to alanine substitution at anchor position 1 (Y1A). Mean ± S.D. *k*_obs_ values are shown for at least three independent experiments. Figure headers (*right*) label the peptide sequence and amino acid anchor positions within the HLA-DR1–binding cleft.

Peptide sequence has been established to be a major factor influencing the observed rate of DM-catalyzed peptide dissociation reactions (8, 9, 45, 46). To assess the impact of individual amino acids on the observed rate, we utilized a fluorescent anisotropy assay that monitored the real-time peptide dissociation from HLA-DR1, a common and well-documented MHC class II molecule, in the presence of titrated concentrations of HLA-DM. Peptide dissociation rates (*k*_obs_) were calculated by fitting each dissociation curve to a single phase exponential decay formula (see “Experimental procedures” for a full description). With a highly stable peptide binder (OPT1) that contains optimal amino acids in the major anchor positions P1, P4, P6, P7, and P9, we observed little to no peptide dissociation over days with HLA-DM concentrations up to 2 μm (Fig. 1*A*). Compared with OPT1, a wide range of DM-mediated peptide dissociation rates was observed when amino acids at anchor positions P1, P4, or P9 had been substituted (Fig. 1, *B–D*). This varied by 1 order of magnitude at P9 for an isoleucine to glutamic acid (I9E) substitution (Fig. 1*B*), 2 orders of magnitude at P4 for a leucine to glycine (L4G) substitution (Fig. 1*C*), and 4 orders of magnitude at P1 for a tyrosine to alanine (Y1A) substitution (Fig. 1*D*). The observed dissociation rates (*k*_obs_) increased with increasing concentrations of HLA-DM, consistent with previous reports (8–10, 28, 32, 47).

**Figure 2.**
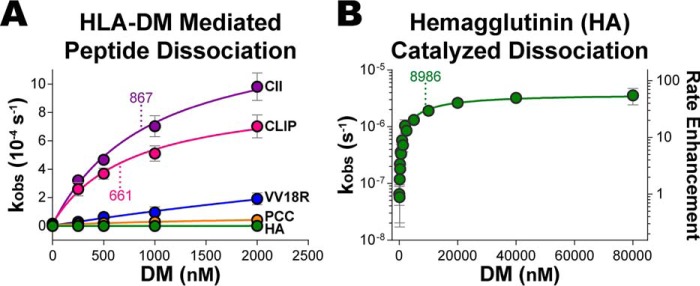
**HLA-DM–catalyzed peptide-dissociation rates saturate.** Dissociation rates (*k*_obs_) of peptides bound to HLA-DR1 were determined by modeling dissociation curves from a fluorescence anisotropy assay to a single-phase exponential decay equation for each DM concentration. *A,* peptide *k*_obs_ rate curves for collagen type-II (CII), CLIP, variola virus RNA-helicase L8R protein (VV18R), pigeon cytochrome *c* (*PCC*), and influenza virus HA-derived peptides. CII and CLIP complexes exhibit saturating *k*_obs_ curves, whereas VV18R, PCC, and HA show linear rate enhancements with up to 2 μm concentration of HLA-DM. *B,* saturation of the observed dissociation rates for the highly stable HA-DR1 complex can be achieved with 80 μm DM. Mean ± S.D. *k*_obs_ values are shown as *solid circles* from data of at least four independent experiments. *Dashed vertical lines* label HLA-DM concentration at the half-maximum peptide dissociation rate.

Highly stable pMHCII complexes have been shown to be relatively resistant to DM activity, as evidenced by the marginal enhancement of peptide dissociation rates, which has led to the proposal of a linear relationship between the observed peptide dissociation rate and DM concentration (6, 9, 28, 45, 47). We evaluated peptide dissociation rates with increasing concentrations of DM and consistently observed upper rate limits with higher concentrations of HLA-DM (Fig. 2). Both the projected maximum dissociation rate and the DM concentration at which the dissociation rate was at half-maximum depended on the peptide sequence (Fig. 2*A*). We further assayed a DR1 complex bound with a highly stable peptide derived from the HA protein of influenza A virus and were able to observe an upper rate limit as DM concentrations approached 80 μm (Fig. 2*B*), suggesting that DM-catalyzed peptide dissociation rates are saturable for all pMHCII complexes given sufficient concentrations of DM. Moreover, this finding supports the conclusion that DM-catalyzed peptide dissociation reactions follow Michaelis-Menten kinetics, as previously reported in early studies (9, 37).

These observations formed the experimental basis for a comparative study of the independent kinetic parameters of DM's enzymatic activity. We formulated a Michaelis-Menten kinetic model of the catalytic reaction involving a rapid steady-state binding of DM to a pMHCII substrate, assembling the DM-pMHCII catalytic complex (Michaelis complex) defined by the equilibrium dissociation constant *K_d_* (equal to *K_m_* in our experimental derivation), followed by the rate-limiting dissociation of the peptide from the catalytic complex defined by the catalytic turnover rate constant, *k*_cat_ (Fig. S2*A*). Consistent with Michaelis-Menten kinetics, the model equates the upper rate limit (maximum) of peptide dissociation with *k*_cat_ and the DM concentration at half-maximum with the Michaelis constant *K_m_*. Moreover, we derived an experimental specific equation to model the Michaelis-Menten reaction scheme, relating the observed peptide dissociation rates (*k*_obs_) from anisotropy assays to the kinetic parameters of the HLA-DM–catalyzed reactions (Fig. S2, *B* and *C*). The derivation describes the catalytic turnover *k*_cat_ as a measure of the stability of peptide bound in the DM-pMHCII catalytic complex, the Michaelis constant *K_m_* as DM-binding affinity of the pMHCII substrate, and the intrinsic peptide dissociation rate *k*_in_ as a measure of the intrinsic stability of the pMHCII complex.

### Peptide-MHCII instability correlates with HLA-DM binding affinity, catalytic turnover, and susceptibility to DM editing

**Figure 3.**
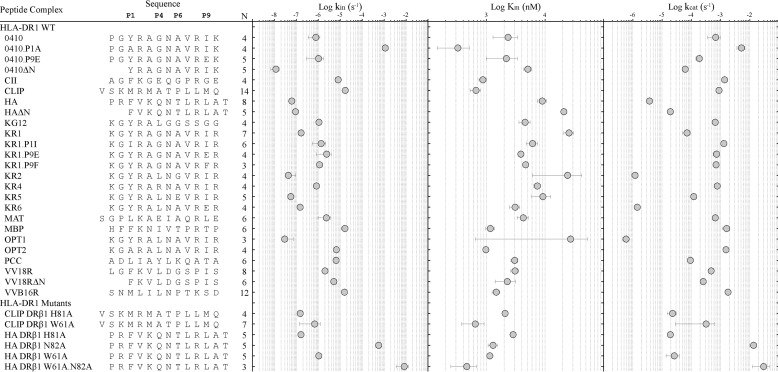
**Kinetic parameters of HLA-DM–catalyzed peptide exchange reactions.** Kinetic parameters for 30 peptide-DR1 complexes were determined by modeling HLA-DM–mediated peptide dissociation rates to an experimentally derived Michaelis-Menten kinetic model (Fig. S2). The parameters spanned 6 orders of magnitude in intrinsic pMHCII stability (*k*_in_), 2 orders of magnitude in DM-binding affinity (*K_m_*), and 5 orders of magnitude for the maximum HLA-DM–mediated peptide dissociation rate (*k*_cat_). Mean ± S.D. values are shown in log scale as *solid circles*. The *leftmost column* identifies the peptide-MHC class II complex, and the peptide sequence aligned to residue occupancy of anchors at position 1 (P1), position 4 (P4), position 6 (P6), and position 9 (P9). Indicated are the number of independent experiments (*N*) performed for each pMHCII. Complexes are grouped by peptides bound to WT or to hydrogen bond-deficient HLA-DR1–binding cleft mutants (Fig. S1*A*). Peptides were acetylated at the N terminus and labeled on the ϵ-amine of lysine residues with Alexa Fluor 488.

A relationship between DM susceptibility and the intrinsic stability of pMHCII complexes has been observed, such that DM activity is increased for less stable peptide complexes (1, 8, 9, 20, 21). Some studies have demonstrated that this relationship is not always observed (11, 27, 28). To more closely explore this relationship, we measured *k*_obs_ rate curves for greater than 30 individual DR1-peptide complexes (Fig. S3) and resolved the kinetic parameters of DM enzymatic activity for each pMHCII substrate to facilitate assessment of kinetic correlations in DM-catalyzed peptide dissociation reactions. The parameters spanned 6 orders of magnitude in intrinsic pMHCII stability (*k*_in_), 2 orders of magnitude in DM-binding affinity (*K_m_*), and 5 orders of magnitude in DM catalytic turnover (*k*_cat_) (Fig. 3).

**Figure 4.**
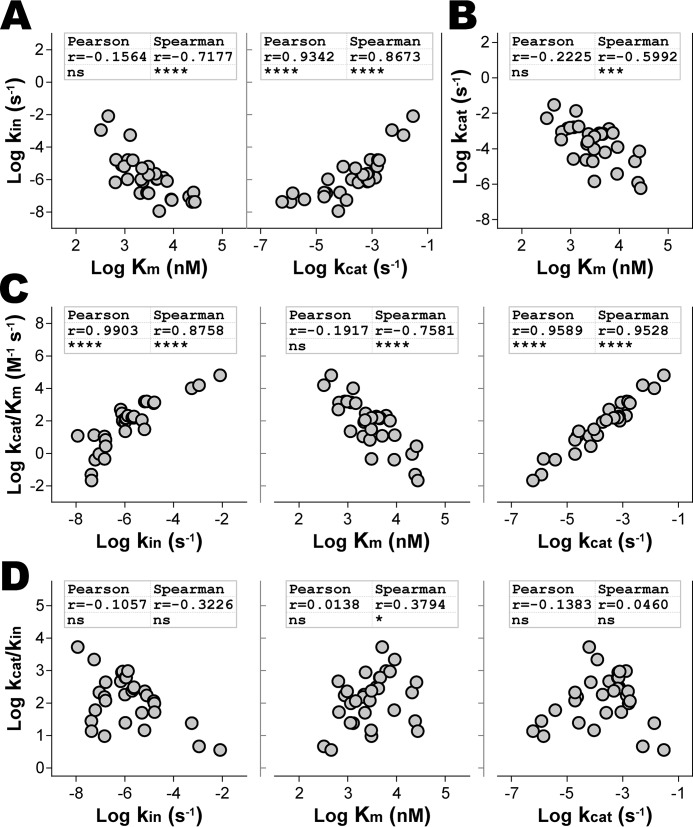
**Correlations in the kinetics of HLA-DM–catalyzed peptide exchange reactions.** Pearson's *r* method was used to assess linear correlations and compared with Spearman's rank method to resolve nonlinear correlations between the kinetic parameters. *A,* intrinsic peptide-MHCII stability (*k*_in_) nonlinearly inverse correlates with DM-binding affinity (*K_m_*) and linearly correlates with DM catalytic turnover (*k*_cat_). *B,* DM catalytic turnover nonlinearly inverse correlates with DM-binding affinity. *C,* peptide-MHCII susceptibility to DM editing (*k*_cat_*/K_m_*) linearly correlates with the intrinsic stability of pMHCII, nonlinearly inverse correlates with DM-binding affinity, and linearly correlates with DM catalytic turnover. *D,* DM catalytic potency (*k*_cat_*/k*_in_) does not correlate with intrinsic pMHCII stability, catalytic turnover, nor considerably with DM-binding affinity. Mean values are shown in log scale as *solid circles* for data of at least three independent experiments. Pearson and Spearman rank correlation null hypothesis was rejected for *p* < 0.05 values. *Inset* shows correlation *r* coefficients and representative *p* values: *, *p* ≤ 0.05; **, *p* ≤ 0.01; ***, *p* ≤ 0.001; ****, *p* ≤ 0.0001; *ns*, not significant (*p* > 0.05).

Pearson correlation analysis was used to assess monotonic linear relationships and Spearman rank correlation was used to evaluate nonlinear relationships between the kinetic parameters. We found a significant inverse Spearman correlation (*r* = −0.7177) between *K_m_* and *k*_in_ (Fig. 4*A, left*), suggesting a nonlinear association between decreased pMHCII stability and increased DM-binding affinity. A robust Pearson correlation (*r* = 0.9342) between *k*_in_ and *k*_cat_ was observed (Fig. 4*A, right*), indicating a direct relationship between the intrinsic stability of the pMHCII and the stability of the peptide bound in the DM-pMHCII catalytic complex. Furthermore, an inverse Spearman correlation (*r* = −0.5992) between DM's catalytic turnover and binding affinity (Fig. 4*B*) suggests a nonlinear coupling between DM-binding affinity and the peptide dissociation rate from the catalytic complex. Together, these results support the idea that a significant number of peptide sequence-specific structural interactions that exist in the resting pMHCII complex are retained in the DM-bound catalytic intermediate.

The specificity constant (*k*_cat_/*K_m_*) was evaluated as a measure of pMHCII susceptibility to DM enzymatic activity. A robust Pearson correlation (*r* = 0.9903) was observed between the specificity constant and intrinsic stability (*k*_in_) (Fig. 4*C, left*). We observed an inverse Spearman correlation (*r* = −0.7581) between the specificity constant and DM-binding affinity (Fig. 4*C, middle*), and a strong Pearson correlation (*r* = 0.9589) with *k*_cat_ (Fig. 4*C, right*). Thus, susceptibility to DM is linearly related to *k*_in_ and to the rate of peptide dissociation from the catalytic complex, but nonlinearly correlated with DM binding affinity. DM catalytic potency (*k*_cat_/*k*_in_) did not correlate strongly with any of the independent kinetic parameters (Fig. 4*D*). Thus, pMHCII intrinsic stability cannot be used to predict the fold-enhancement of peptide dissociation rates in the presence *versus* absence of DM. Overall, however, the findings indicate that the pMHCII intrinsic stability correlates strongly with DM-binding affinity, the rate of peptide dissociation from the catalytic complex, and catalytic specificity.

### Peptide interactions in the N terminus region of the MHCII cleft are retained in the DM-pMHCII catalytic intermediate

The pH 5.5 DM-DR1 co-crystal structure is presumed to depict the active DM-pMHCII catalytic complex (41). Structural rearrangements in DR1 proximal to the DM-binding site preclude occupancy of the peptide N terminus up to and including the dominant P1 anchor position. If this structure accurately represents the DM-pMHCII catalytic complex, then interactions between the peptide N terminus and MHCII should not influence the stability of the catalytic complex (*k*_cat_) because these interactions do not exist in the catalytic complex (34, 41, 42).

**Figure 5.**
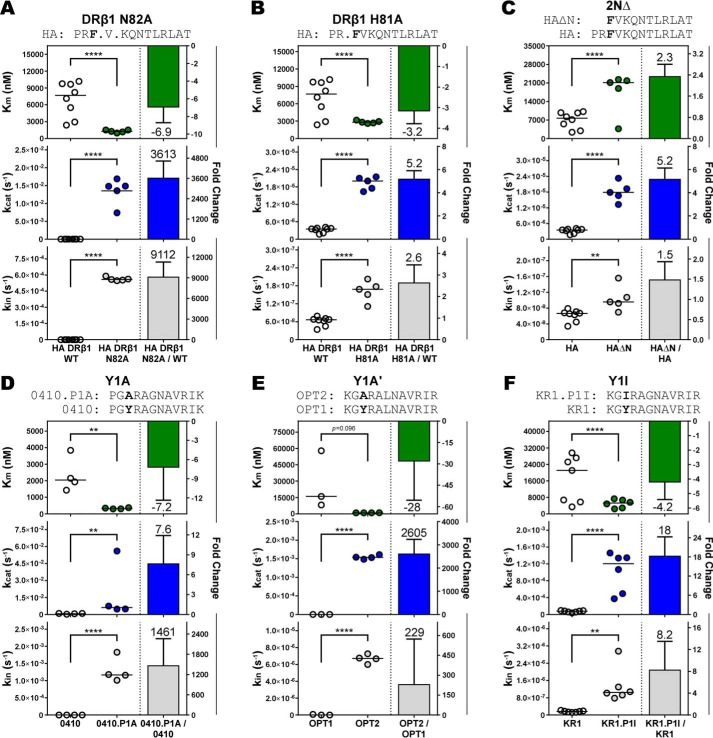
**N terminus peptide interactions with the MHCII are retained in the DM-pMHCII catalytic intermediate.** The ratio of the kinetic values between homologous peptide-DR1 complexes were computed to determine the relative change in the kinetic parameters. Complexes varied by a single chemical or structural feature within the peptide N terminus region of the MHCII-binding cleft. Homologous pMHCII complexes differed by: (*A*) a DRβ1 N82A mutant that eliminates two hydrogen bonds flanking the P2 anchor; (*B*) a DRβ1 H81A mutant that eliminates a single hydrogen bond N-terminal proximal to the P1 anchor; (*C*) truncation of the peptide P-2 and P-1 residues (2NΔ) that eliminates three hydrogen bonds N-terminal to the P1 anchor; and (*D* and *E*) amino acid occupancy of the P1 anchor pocket: (*D*) tyrosine to alanine (Y1A), (*E*) an additional tyrosine to alanine (Y1A′); and (*F*) tyrosine to isoleucine substitution (Y1I). N terminus peptide interactions significantly altered DM-binding affinity (*K_m_*), the rate of peptide dissociation from the catalytic complex (*k*_cat_), and the intrinsic stability of the pMHCII (*k*_in_). Mean ± S.D. fold-change shown in *bars* for data of at least three independent experiments with mean values given over/under *error bars* and *negative signs* denoting decreases in magnitude. Distributions and values of the kinetic parameters shown on *left* of panels with *horizontal lines* denoting distribution means. Figure titles identify peptide sequences with *bold* labeling the varied chemical or structural feature. Full points inserted between residues identify the location of eliminated hydrogen bonds in HLA-DR1 mutants. Independent two-sample Student's *t* test: *, *p* ≤ 0.05; **, *p* ≤ 0.01; ***, *p* ≤ 0.001; ****, *p* ≤ 0.0001; *ns*, not significant (*p* > 0.05).

The Asn-82 residue of the DRβ1 domain forms highly-conserved bidentate hydrogen bonds with the peptide main chain straddling the P2 residue in MHCII-peptide complexes (Fig. S1*A*) (31, 32, 48). An asparagine to alanine mutation (N82A) was observed to profoundly increase *k*_in_ for DR1-HA peptide complexes (Fig. 5*A*), underscoring the importance of this feature to the intrinsic stability of the pMHCII complex (31–33, 40). This mutation in DR1-HA significantly decreased *K_m_*, denoting a higher DM-binding affinity. Importantly, the N82A substitution markedly enhanced peptide dissociation from the catalytic complex (*k*_cat_) >3600-fold, suggesting that the two hydrogen bonds mediated by Asn-82 are retained in the active DM-pMHCII catalytic complex, serving as potent stabilizers of the catalytic intermediate.

We explored the possibility that additional peptide N terminus interactions with the MHCII may be retained in the catalytic complex. We compared a pair of DR1-HA complexes varying in a histidine to alanine mutation at DRβ1 position 81 (DRβ1 H81A) that eliminates a single hydrogen bond between the MHCII and the peptide main chain on the carbonyl group of the P-1 residue (Fig. S1*A*). Consistent with the previous observation, DRβ1 H81A decreased the intrinsic stability of the pMHCII (*i.e.* greater *k*_in_) and it increased DM-binding affinity (*i.e.* reduced *K_m_*) (Fig. 5*B*). Notably, we observed an ∼5-fold enhancement in peptide dissociation from the catalytic complex (*k*_cat_), suggesting that the His-81–mediated hydrogen bond was also retained in the catalytic complex. The impact of truncation of two N-terminal residues (2NΔ) of the HA peptide, functionally removing three hydrogen bonds between the MHCII and the peptide main chain and occupancy of the P-2 and P-1 residues (Fig. S1, *A* and *B*) was evaluated. This resulted in a ∼5-fold increase in *k*_cat_ (Fig. 5*C*), indicating that the two N-terminal peptide residues influence the stability of the catalytic complex.

These results suggested that peptide occupancy of the P1 anchor pocket might be conserved in the catalytic complex. The P1 anchor is the deepest and most hydrophobic pocket within the binding cleft of HLA-DR1, optimally binding large aromatic residues (48, 49). Peptide occupancy of the anchor dominantly influences the intrinsic stability of the complex and plays a substantial role in the peptide-binding motif of HLA-DR1. To test whether peptide occupancy of the P1 anchor is retained in the catalytic complex, we compared DR1-peptide complexes varying in the identity of the P1 residue. In the context of the 0410 peptide, an optimal tyrosine to a small aliphatic alanine substitution at P1 (Y1A) resulted in a considerable decrease in the intrinsic stability of the pMHCII (higher *k*_in_), increased DM-binding affinity (lower *K_m_*), and a significant ∼8-fold enhancement in *k*_cat_ (Fig. 5*D*). In the context of the OPT1 peptide, we observed a lesser impact on the stability of the pMHCII complex by the alanine substitution (Y1A′), yet an increased >2600-fold enhancement in *k*_cat_ (Fig. 5*E*). This observation suggests that the energetic contribution to the kinetics of DM activity by specific anchor-pocket interactions is influenced by the overall sequence of the bound peptide. Nonetheless, these results support the conclusion that the identity of the peptide P1 anchor residue influences the stability of the DM-pMHCII catalytic complex. This conclusion was further supported by a tyrosine to isoleucine substitution (Y1I) that enhanced *k*_cat_ by 18-fold (Fig. 5*F*). Altogether, the results provide strong evidence that interactions between DR1 and the N terminus of bound peptides can have a considerable influence on the rate of peptide dissociation from the catalytic intermediate, supporting the conclusion that these elements are conserved in the DM-pMHCII catalytic complex.

### Peptide interactions with the P4 and P6 anchor pockets in the central region of the MHCII-binding cleft predominantly stabilize the DM-pMHCII catalytic intermediate

**Figure 6.**
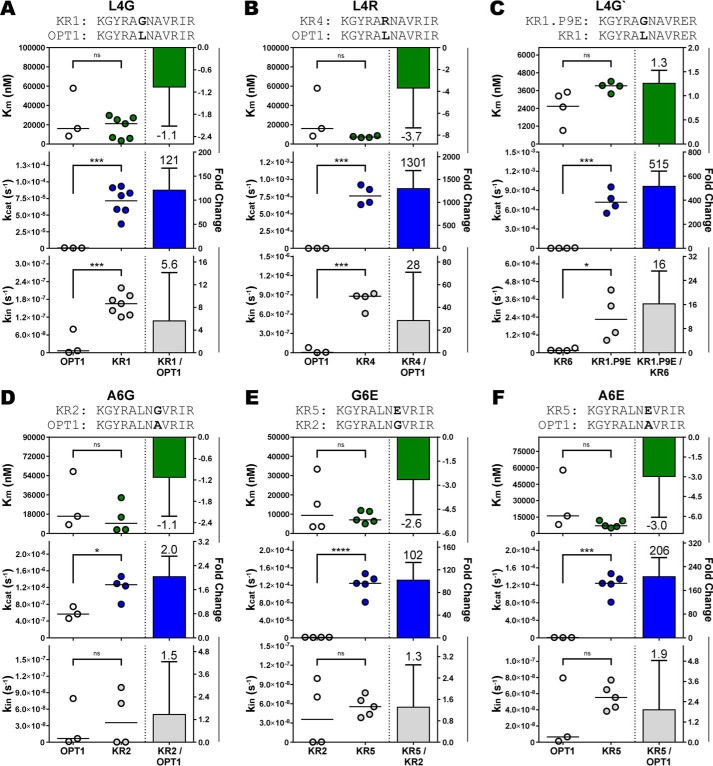
**Peptide interactions in the central region of the MHCII-binding cleft with P4 and P6 anchor pockets predominantly stabilize the DM-pMHCII catalytic intermediate.** The relative change in the kinetics of HLA-DM–catalyzed peptide exchange reactions were determined by computing the ratio of the kinetic values between homologous peptide-DR1 complexes that varied by amino acid occupancy of the P4 or P6 anchor pockets. Homologous pMHCII complexes differed in P4 occupancy by (*A*) leucine to glycine (L4G), (*B*) leucine to a charged arginine (L4R), (*C*) a second leucine to glycine substitution (L4G′); or P6 occupancy by (*D*) alanine to glycine (A6G), (*E*) glycine to a charged glutamic acid (G6E), and (*F*) alanine to a charged glutamic acid substitution (A6E). Mean ± S.D. fold-change shown in *bars* for data of at least three independent experiments with mean values given over/under *error bars* and *negative signs* denoting decreases in magnitude. Distributions and values of the kinetic parameters are shown on *left of the panels* with *horizontal lines* denoting distribution means. Figure titles identify the peptide sequences with *bold* labeling the chemical feature under consideration. Independent two-sample Student's *t* test: *, *p* ≤ 0.05; **, *p* ≤ 0.01; ***, *p* ≤ 0.001; ****, *p* ≤ 0.0001; *ns*, not significant (*p* > 0.05).

The contribution of interactions within the central region of the MHCII-binding cleft on the DM catalytic mechanism was assessed by evaluating the relative change in kinetic parameters with single amino acid substitutions in the peptide P4 and P6 anchor positions. The P4 pocket in HLA-DR1 is hydrophobic and relatively shallow, preferring moderately sized aliphatic residues (48, 49). The substitution of an optimal leucine at P4 in the OPT1 peptide with a less favorable glycine (L4G) reduced the intrinsic stability of the complex (increased *k*_in_), but had no significant impact on DM-binding affinity (*K_m_*) (Fig. 6*A*). The glycine substitution had a marked >120-fold increase in *k*_cat_, however, indicating that interactions involving the P4 anchor help to stabilize the DM-DR1 catalytic complex. The P4 pocket interaction was further destabilized by replacing leucine with arginine (L4R), a charged residue that is chemically incompatible with the properties of the P4 pocket. This substitution resulted in a marked >1300-fold increase in *k*_cat_, but again no significant effect on DM-binding affinity was observed (Fig. 6*B*). These results were additionally mirrored in a separate leucine to glycine substitution (L4G′) that was evaluated in the context of an OPT1 peptide that has an unfavorable glutamic acid residue at the P9 anchor position (Fig. 6*C*). Notably, the glycine substitution at P4 with complexes containing a disfavored P9 anchor had a greater impact on *k*_cat_ (510-fold increase) than those containing an optimal P9 residue (Fig. 6, *A* and *C*), again demonstrating that the energetic contributions of specific anchor-pocket interactions can be substantially modified by interactions elsewhere in the peptide-binding cleft.

The P6 pocket in HLA-DR1 is hydrophobic and the most shallow of the dominant pockets, preferring small aliphatic residues (48, 49). Replacement of an optimal alanine at P6 in OPT1 with an achiral glycine (A6G) resulted in a modest 2-fold increase in *k*_cat_ with no significant impact in *k*_in_ nor *K_m_* (Fig. 6*D*). Replacement with the chemically unfavorable glutamic acid, however, substantially increased *k*_cat_ by >100- or 200-fold, respectively (Fig. 6, *E* and *F*). Altogether, the results of substitutions in P4 and P6 anchor residues indicate that interactions in the central region of the MHCII peptide-binding cleft have negligible influence on DM-binding affinity but considerable impact on the stability of the DM-pMHCII catalytic complex.

### Distal C terminus peptide interactions with the MHCII are sensed at the DM-pMHCII binding interface

**Figure 7.**
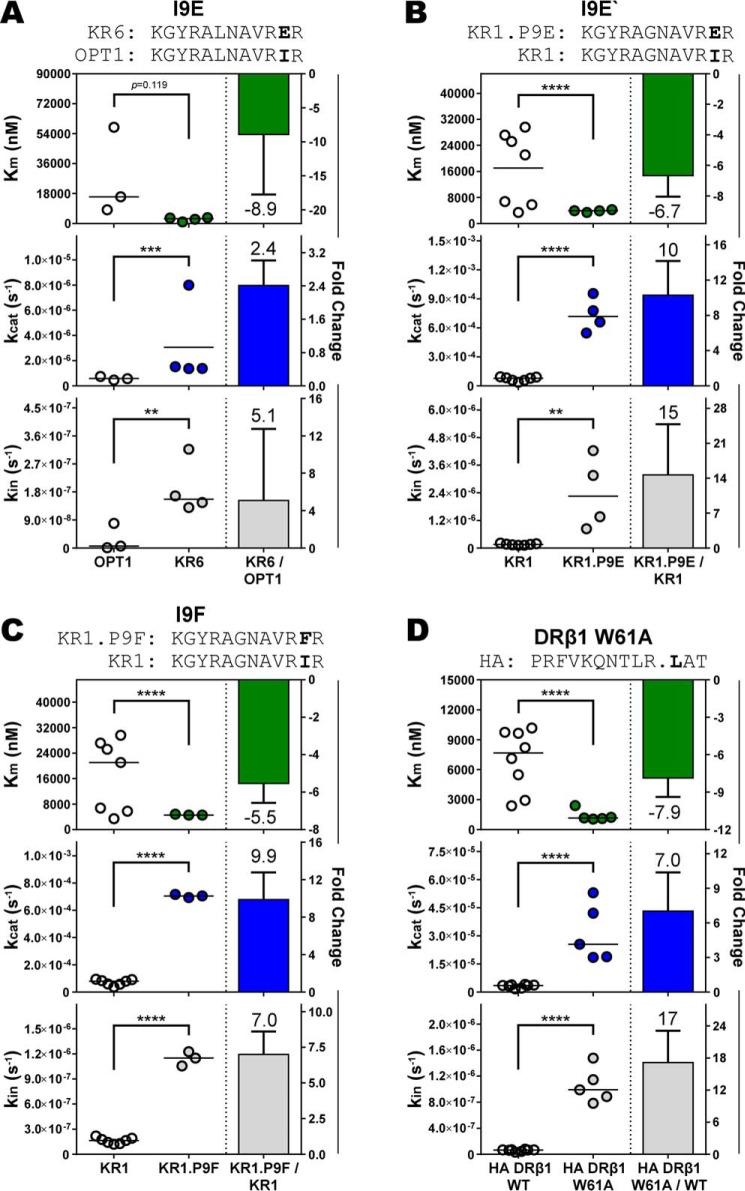
**C terminus peptide interactions with the MHCII are distally communicated to the DM-pMHCII binding interface.** The ratio of the kinetic values between homologous peptide-DR1 complexes were computed to determine the relative change in the kinetic parameters. Complexes varied by a single chemical or structural feature within the peptide C terminus region of the MHCII-binding cleft. Homologous pMHCII complexes differed by (*A-C*) amino acid occupancy of the P9 anchor pocket by (*A*) isoleucine to a charged glutamic acid (I9E), (*B*) a second isoleucine to charged glutamic acid (I9E′), (*C*) isoleucine to a bulky phenylalanine substitution (I9F); or by (*D*) a DRβ1 W61A hydrogen-bond mutant that eliminates a single hydrogen bond N-terminal proximal to the P9 anchor pocket. C terminus peptide interactions significantly altered DM-binding affinity (*K_m_*), the rate of peptide dissociation from the catalytic complex (*k*_cat_), and the intrinsic stability of the pMHCII (*k*_in_). Mean ± S.D. fold-change are shown in *bars* for data of at least three independent experiments with mean values given over/under *error bars* and *negative signs* denoting decreases in magnitude. Distributions and values of the kinetic parameters are shown on the *left of panels* with *horizontal lines* denoting distribution means. Figure titles identify peptide sequences with *bold* labeling the chemical or structural feature. *Full points* inserted between residues identify the location of eliminated hydrogen bonds in HLA-DR1 mutants. Independent two-sample Student's *t* test: *, *p* ≤ 0.05; **, *p* ≤ 0.01; ***, *p* ≤ 0.001; ****, *p* ≤ 0.0001; *ns*, not significant (*p* > 0.05).

The impact of interactions at the peptide C terminus on the kinetic parameters of DM activity was also evaluated. The P9 pocket in DR1 is hydrophobic and the second largest pocket, with a preference for moderate sized aliphatic residues (48, 49). We compared a complex with an aliphatic isoleucine residue to a homologous complex with a charged acidic glutamic acid at P9 (I9E). This substitution resulted in a decrease in the intrinsic stability (increased *k*_in_) of the pMHCII and an increase in the catalytic turnover *k*_cat_ (Fig. 7*A*). A trending ∼9-fold decrease (*p* = 0.119) in *K_m_* was observed, implying increased DM-binding affinity, although the effect did not reach the level of statistical significance. However, in comparing two additional pairs of DR1 complexes containing related peptides in which an unfavorable charged acidic glutamic acid (I9E′) or a bulky aromatic phenylalanine residue (I9F) replaced an optimal isoleucine in P9, we observed significant ∼7- and 6-fold increases, respectively, in DM-binding affinity (Fig. 7, *B* and *C*). These substitutions also resulted in increases in *k*_in_ and *k*_cat_, reflecting destabilization of both the resting and catalytic complexes.

The effect of interactions at the C terminus of the bound peptide on DM-binding affinity was further addressed by evaluating a tryptophan to alanine substitution at amino acid position 61 of the β1 domain (W61A) of DR1. This mutation, which eliminates a single hydrogen bond with the main chain of the bound peptide between P8 and P9 (Fig. S1*A*), resulted in a significant ∼8-fold increase in DM-binding affinity and concomitant increases in *k*_in_ and *k*_cat_ (Fig. 7*D*) as compared with the WT DR1-HA complex. The finding that *K_m_* is influenced by interactions between the MHCII and peptide C terminus provides evidence that chemical and structural features distal to the DM-binding site have a long-range effect on DM-binding affinity.

## Discussion

We developed a real-time fluorescent anisotropy assay to monitor experimentally irreversible peptide dissociation from peptide-DR1 complexes and observed rate saturation in the presence of increasing concentrations of DM. The DM concentrations required to approach saturation varied considerably among >30 distinct pMHCII complexes analyzed. This behavior was consistent with an enzyme-like catalytic mechanism that could be modeled by a Michaelis-Menten kinetic reaction scheme. A mathematical model was derived that nicely fit the observed experimental results, providing a method to determine the kinetic parameters of DM-catalyzed peptide dissociation reactions for a large number of pMHCII complexes. Previous studies have examined the kinetics of DM activity for a small number of pMHCII complexes (9, 10, 37, 42, 50). The methodologies were unable to delineate the independent contributions of DM-binding affinity and catalytic turnover to the overall catalytic mechanism, nor the contributions of discrete structural and chemical determinants to the kinetic parameters. We quantified a breadth of kinetic values spanning 6-logs in intrinsic peptide-MHCII stability, 2-logs in DM-binding affinity, and 5-logs in DM catalytic turnover rates.

In an early study, DM was observed to enhance the rate of peptide dissociation to an extent directly proportional to the intrinsic rate of peptide dissociation from HLA-DR1 (8). Many studies have since reinforced the conclusion that DM preferentially edits pMHCII complexes with low intrinsic stability, favoring the presentation of the most stable complexes for recognition by T cells (9, 12, 20, 21, 51, 52). Our data support this conclusion in that we observe a strong direct correlation (Pearson *r* = 0.9903) between pMHCII stability and susceptibility to DM editing, as gauged by the specificity constant *k*_cat_/*K_m_*, which quantifies the catalytic efficiency of DM when the pMHCII substrate concentration is much less than *K_m_* (Fig. 4). We observed a direct linear correlation between the intrinsic stability of pMHCII and DM catalytic turnover, and an indirect nonlinear correlation with DM-binding affinity, demonstrating that reduced peptide complex stability results in monotonic increases to both DM catalytic turnover and DM-binding affinity. However, there are clear examples where intrinsic stability does not predict the degree of susceptibility to DM editing (27, 28). In a recent study with HLA-DQ6, examples of high stability peptide complexes were identified that were sensitive to DM editing (11). A high level of pMHCII intrinsic stability was found to be necessary but not sufficient to confer resistance to DM activity. Interestingly, we do not find a statistically significant correlation between complex stability and DM catalytic potency *k*_cat_/*k*_in_, as defined by the ratio of the catalyzed peptide dissociation rate *versus* the intrinsic peptide dissociation rate. The intrinsic stability of pMHCII complexes does not then predict the fold-enhancement in peptide dissociation rates observed in the presence of DM.

Several studies have provided evidence that interactions involving the peptide N terminus are key determinants of DM susceptibility (27, 39, 40). The results presented herein are consistent with these studies in that the data show that instability of the complex is strongly correlated with DM-susceptibility (*k*_cat_/*K_m_*) and instability of the N-terminal segment of the bound peptide can have a large impact on DM-binding affinity and catalytic turnover (Fig. 5).

A prevailing model of DM catalysis has further proposed that partial dissociation of the peptide N terminus including the P1 anchor is necessary for DM to bind pMHCII (41, 42), arguing that the propensity of a peptide to partially dissociate from an MHCII molecule is the critical determinant of DM susceptibility. By contrast, our results suggest that interactions involving the peptide N terminus are preserved in the DM-bound catalytic complex, and that these interactions can have a major impact on the stability of peptide bound in the catalytic complex. The observed effects on *k*_cat_ of N-terminal peptide truncations, P1 anchor substitutions, and mutational disruption of conserved hydrogen bonds involving the peptide N terminus are all inconsistent with a model in which DM binds only to pMHCII complexes in which peptide has partially dissociated at the N terminus.

The prevailing model was in part deduced from the DM-DR1 co-crystal structure that was solved at pH 5.5 and 6.5 (41). The structures were resolved for an HA peptide disulfide linked at the P6 anchor and truncated at P-2, P-1, and P1 residues. Both structures showed DRα W43 rotated out of the peptide-binding cleft, forming a hydrogen bond with DMα Asn-125. The DRα 3_10_-helix was reoriented and merged with neighboring residues, forming an extended α-helix. The two structures diverged in the structural rearrangement of some key residues. Notably, the pH 5.5 structure showed DRα Phe-51 and DRβ Phe-89 repositioned into the P1 pocket, thereby precluding occupancy with the dominant P1 anchor side chain. It was reasoned that this conformational state could only be achieved by disruption of hydrogen bonds and vacancy of peptide side chains from the peptide N terminus to the P1 anchor, forming the foundation for a model in which partial dissociation of the peptide N terminus is required for DM binding.

Compared with resting pMHCII crystal structures, there are major alterations to the peptide conformation and MHCII interactions in the pH 5.5 co-crystal that suggest it may not represent the functional DM-DR1 catalytic complex. Of note, the crystal lacks the conserved hydrogen bond network between the peptide main chain and flanking DRα1 and DRβ1 helices that are observed in all MHCII crystal structures (17, 48, 49, 53, 54). Electronic densities are observed for P5–P11 peptide residues but not for P2–P4. The P6 residue (tethered position) is shown elevated above the binding cleft. P5 is atypically directed away from the cleft. In addition, the peptide backbone is not in the polyproline type II conformation that is characteristic of a bound peptide. Taken together, these observations suggest that the pH 5.5 co-crystal structure may better represent an inactive late intermediate that depicts DM bound to a peptide-free, empty DR1 molecule rather than the functional DM-pMHCII catalytic complex (24, 25).

**Figure 8.**
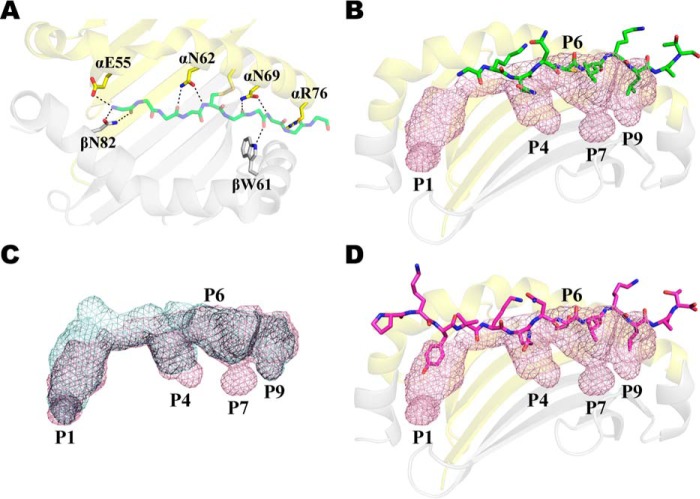
**The DM-DR1 pH 6.5 co-crystal structure models the active DM-pMHCII catalytic complex.** The pH 6.5 cocrystal shows a peptide in a polyproline II main chain conformation with full side chain occupancy of the anchor pockets and electronic densities of all peptide residues. *A,* the conserved hydrogen bond network is intact for all peptide residues. DRαE55, which forms a H_2_O-meditated bond with DRβN82 in the pH 5.5 structure, is shown in an intermediate position forming a hydrogen bond with the peptide N terminus. The DRβN82 bidentate hydrogen bonds are shown in their resting position flanking the P2 residue. *B,* the truncated and tethered HA peptide (*green*) is shown occupying the available anchor pockets (*pink mesh*). *C,* anchor pockets in DM-DR1 pH 6.5 structure (*pink mesh*) *versus* the resting HLA-DR1 (*cyan mesh*) structure. The major anchor pockets largely retain their depth and dimensions when compared with the resting DR1 structure (overlapping *black mesh*). *D,* full-length HA peptide (magenta) from the resting DR1 crystal structure is superimposed in the anchor pockets of the pH 6.5 DM-DR1 structure. Figures were generated with PyMOL (Schrödinger, LLC) using the DM-DR pH 6.5 cocrystal (PDB ID 4GBX) and resting DR1 crystal structure (PDB ID 1DHL). HLA-DR1–binding cleft is shown in ribbon diagram (DRα, *yellow*; DRβ, *gray*) bound to a HA peptide shown in stick representation.

The data presented herein are consistent with the DM-DR1 structure at pH 6.5 as a depiction of an active catalytic complex (41). In contrast to the pH 5.5 structure, the pH 6.5 co-crystal profiles a peptide with a polyproline II main chain conformation, side chain occupancy of the anchor pockets with existing anchor residues, and electronic densities throughout P2–P11 peptide residues (Fig. 8). The structure similarly shows DRα Trp-43 rotated away from the P1 pocket and interacting with DMα Asn-125. Notably, despite the absence of the peptide P1 residue, DRα Phe-51 and DRβ Phe-89 residues are not yet positioned in the P1 pocket, but are found in an intermediate position between that of the resting DR1 and pH 5.5 DM-DR1 structures. Similarly, DRα Glu-55 is intermediately positioned allowing DRβ Asn-82 to maintain the bidentate hydrogen bonds with the peptide main chain that straddle the P2 residue (Fig. 8*A*). In addition, the P1 anchor pocket is not occluded by the conformational change associated with DM binding (Fig. 8*B*). Instead, the dimensional and structural features are largely retained when compared with the P1 pocket of the resting DR1 complex, including the capacity to bind large hydrophobic amino acid residues (Fig. 8, *C* and *D*). Together, this strongly suggests that the conformational state of the DM-DR1 co-crystal structure at pH 6.5 may better represent the functional catalytic complex. In this model, DM can bind pMHCII with full occupancy of the peptide-binding cleft, and interactions involving the peptide N terminus can influence the stability of the catalytic complex.

Our data further imply that peptide interactions throughout the binding cleft impact DM susceptibility. Analysis of homologous complexes with substitutions at the peptide P4 anchor position had a major impact on *k*_cat_, but did not generally impact DM-binding affinity (Fig. 6). These results were mirrored with substitutions at the peptide P6 position, suggesting that interactions involving the central region of the MHCII-binding cleft predominantly serve to stabilize the catalytic intermediate. With P9 anchor substitutions, *k*_cat_ was also significantly impacted (Fig. 7), together suggesting that peptide anchor residues throughout the length of the peptide-binding cleft influence the stability of peptide bound in the functional catalytic complex.

Importantly, our data show that the magnitude of the impact on the kinetic parameters by individual interactions between peptide and MHCII depends on the overall context of the peptide sequence. For a tyrosine to alanine substitution at the P1 anchor position, the fold-enhancement in *k*_cat_ was markedly influenced by the identity of the amino acid at the P4 anchor position (Fig. 5), demonstrating that distal interactions can influence the energetic contributions of remote structural elements in the catalytic intermediate. This observation is consistent with studies suggesting that the global conformational state of the pMHCII is a determinant of DM susceptibility (20, 28, 43, 44, 55). This notion was exemplified by interactions involving the peptide C terminus that resulted in a significant impact to DM-binding affinity. Substitutions at the P9 anchor position or disruption of a conserved hydrogen bond between DR1 and the peptide C terminus altered *K_m_*, suggesting that interactions at the distal end of the peptide-binding cleft had a long-range effect on the conformation dynamics of the DM-binding interface. This conclusion is consistent with separate studies showing that structural features along the entire peptide-binding cleft are relevant to DM peptide editing effects (20, 28).

It is likely that DM catalysis involves very dynamic and concerted structural rearrangements that are initiated upon DM binding to a pMHCII. Peptide sequence is a significant factor in determining susceptibility to DM action. The P1 anchor is dominant in the DR1 peptide-binding motif, but other anchor positions play a more important role in other MHCII molecules. MHCII alleles have varying chemical features in the binding cleft that define both the preference of distinct peptide residues at anchor sites and the relative contribution of each anchor to the stability of the MHCII. It is likely that DM susceptibility varies based on MHCII polymorphisms. Indeed, recent studies report that type-1 diabetes-associated HLA-DQ molecules are relatively resistant to DM action (56, 57). Further studies are needed to investigate the kinetics and mechanism of DM function with MHCII molecules that differ in peptide-binding specificity. Our data show that the magnitude of contribution of specific peptide interactions to the kinetic parameters is influenced by the entire peptide sequence, consistent with the idea that the global conformational dynamics in pMHCII may be the defining determinant of DM action.

## Experimental procedures

### Cell lines

*Drosophila* S2 cell line expressing soluble recombinant HLA-DM (DMA*0101/DMB*0101) was kindly provided by Dr. Elizabeth Mellins (Stanford University), and generated as previously described (6). Briefly, cells were cotransfected with pRmHA3-induced expression vector (Addgene) encoding cDNA from the extracellular domains of the DMα chain fused to a C-terminal FLAG peptide and DMβ chain fused to a C-terminal SV40 large T-antigen epitope. *Drosophila* S2 cell lines expressing soluble recombinant WT or mutant HLA-DR1 (DRA*0101/DRB1*0101) were generated as previously described (33). Briefly, cells were cotransfected with pRmHA3-induced expression vector encoding cDNA from the extracellular domains of the DR1α chain fused to a C-terminal FLAG peptide and unlabeled DR1β chain. *Drosophila* S2 cell lines were maintained in Schneider's *Drosophila* medium supplemented with 10% heat-inactivated fetal bovine serum in a 28 °C cell culture incubator.

Human embryonic kidney (HEK) 293T cell line expressing soluble recombinant HLA-DR1 (DRA*0101/DRB1*0101) was generated as previously described (56). Briefly, cells were lentiviral transduced with pWPI constitutive expression vector encoding the cDNA from the extracellular domains of unlabeled DR1α chain and DR1β chain fused to a C-terminal His_6_ peptide and interchain-linked with the T2A ribosome skip sequence. HEK 293T cells were maintained in Dulbecco's modified Eagle's medium supplemented with 10% heat-inactivated fetal bovine serum, 100 units/ml of penicillin, 100 μg/ml of streptomycin, 2 mm l-glutamine, 100 μm nonessential amino acids, 1 mm sodium pyruvate, and 55 μm 2-mercaptoethanol in a humidified cell culture incubator containing 5% CO_2_ at 37 °C.

### Protein purification

Soluble HLA-DM and HLA-DR1 proteins produced in *Drosophila* S2 cell lines were purified as previously described (33). Briefly, expression of HLA-DM or HLA-DR1 was induced by 1 mm CuSO_4_ in BaculoGold Max-XP (BD Biosciences) serum-free cell culture media. After 7 days, supernatant was cleared of cellular debris by centrifugation and 0.45-μm vacuum filtration (Corning). Filtrate was concentrated in a stirred 30-kDa MWCO PES ultrafiltration concentrator (Millipore) under N_2_ pressure at 4 °C. Concentrate was buffer exchanged on a HiPrep 26/10 desalting column (GE Healthcare) into PBS prior to protein isolation. Soluble HLA-DR1 produced in HEK 293T cells was purified as previously described (56). Briefly, supernatant from cells constitutively expressing HLA-DR1 was cleared of cellular debris by centrifugation and 0.45-μm vacuum filtration (Corning). HLA-DR1 was concentrated on an in-house IMAC column packed with nickel-nitrilotriacetic acid His tag purification resin (Roche Applied Science) prior to isolation. Proteins were isolated on in-house affinity columns packed with M2 (anti-FLAG peptide) affinity gel (Sigma) for HLA-DM, or Protein A-Sepharose resin (GE Healthcare) covalently bound to L243 antibody for HLA-DR1 as previously described (58). Aggregates were removed on a Superdex 200 gel filtration column (GE Healthcare). Chromatography was done on an ÄKTA fast protein LC (FPLC) system (Amersham Biosciences) using standard protocols. Proteins were quantified by averaging the concentration derived by UV absorbance at 280 nm using the calculated protein sequence-specific extinction coefficient, and sandwich ELISA using in-house protein standards. Protein aliquots were functionally evaluated by a fluorescence anisotropy peptide dissociation assay for consistency in measurements, assessed for purity by Coomassie stain, and identity by Western blotting. Protein stocks were concentrated on 30-kDa MWCO spin concentrators (Amicon) and snap frozen in liquid N_2_ prior to −80 °C storage.

### Peptide synthesis and fluorophore labeling

N-terminal acetylated peptides were synthesized in-house or commercially acquired from Celtek Peptides (Celtek Bioscience). In-house peptides were synthesized on a PS3 peptide synthesizer (Protein Technologies) using standard solid-phase Fmoc (*N*-(9-fluorenyl)methoxycarbonyl) chemistry. Briefly, synthesis was initiated on 25 μmol of preloaded Wang resin. Peptide chains were cleaved from resin with 82.5% TFA, 5% thioanisole, 5% H_2_O, 5% phenol, and 2.5% EDT. Cleaved peptides were precipitated overnight in diethyl ether and air dried prior to purification. Crude peptides were resuspended in DMSO and purified on an Agilent 1200 series by RP-HPLC using a Jupiter 4μ Proteo 90Å C12 (Phenomenex) column in a multistep gradient elution from 10 to 60% solvent B (acetonitrile, 0.09% TFA). Peptides synthesized or purchased from Celtek Peptides were verified for >95% purity by RP-HPLC and molecular weight by MALDI-MS. Peptides were quantified by UV 205 nm absorbance using the calculated peptide main chain-specific extinction coefficient. Peptide stocks were lyophilized and stored at −80 °C.

Peptides were labeled on the ϵ-amine of lysine residues with Alexa Fluor 488 5-TFP (Molecular Probes) following a modified manufacturer's protocol, and purified as described (33). Briefly, peptide and fluorophore were mixed in binding buffer (100 mm triethylamine in DMSO) at a 4:1 molar ratio of peptide to fluorophore. The reaction proceeded on a rotator in the dark at room temperature for 1 h and overnight at 4 °C. Labeled peptides were separated from unlabeled on an Agilent 1200 series by RP-HPLC using a Vydac 218TP52 C18 column (Grace). Fractions containing labeled peptides were identified by retention-time shift and fluorophore UV 494 nm absorbance peak. Fluorophore-labeled peptides were verified for molecular weight by MALDI-MS. Peptides were quantified by averaging the concentration derived from UV absorbance at 205 nm using the calculated peptide main chain-specific extinction coefficient, and fluorophore UV absorbance at 494 nm. Labeled stock peptides were lyophilized and stored at −80 °C.

### Fluorophore-labeled pMHCII complex formation

Labeled peptide-MHCII complexes were generated as previously described (33). Briefly, a 50:1 molar ratio of labeled peptide to HLA-DR1 in loading buffer (250 mm citrate-phosphate buffer, pH 5.0, 150 mm NaCl) were incubated on a rotator in the dark at 37 °C. After 24–72 h, endogenous peptide exchange was terminated by pH neutralization (Tris-HCl, pH 8.0) and temperature reduction (4 °C). Labeled pMHCII complexes were separated from aggregates and unlabeled complexes on a Superdex 75 10/300 GL gel filtration column (GE Healthcare) using a Shimadzu HPLC System equipped with a RF-10AxL florescence detector. Fractions containing labeled peptide-DR1 complexes were identified by retention-time shift and fluorophore UV 535 nm peak emission. Labeled complexes were quantified by averaging the concentration derived from UV absorbance at 280 nm using the calculated protein sequence-specific extinction coefficient, and fluorophore UV absorbance at 494 nm. Protein stocks were concentrated on 30-kDa MWCO spin concentrators (Amicon) and snap frozen in liquid N_2_ prior to −80 °C storage.

### Fluorescence anisotropy peptide-dissociation assay

Real-time HLA-DM–catalyzed peptide dissociation reactions were measured by fluorescence anisotropy. Anisotropic peptide dissociation curves were generated for fluorophore-labeled peptide-DR1 complexes (50 nm) with DM concentrations up to 90 μm and 400-fold excess unlabeled, high binding-affinity peptide (20 μm) to prevent rebinding of signal (fluorophore-labeled) peptide. Assayed concentrations of HLA-DM were chosen to extend above *K_m_*, achieve reaction equilibrium within 14 days, and produce a curvature of observed dissociation rates approaching saturation. A prereaction mix was prepared at room temperature in citrate/phosphate buffer (250 mm citrate, 150 mm NaCl, pH 5.0) containing HLA-DM, unlabeled competitor peptide, and 25 μl of mineral oil (surface overlay) to prevent evaporation. Prereactions were incubated at 37 °C for 15 min prior to initiation by addition of 1 μl of labeled peptide-DR1 complexes. Anisotropy of peptide dissociation from HLA-DR1 complexes was recorded over a 1–14 day span at 37 °C and terminated when at least one peptide dissociation curve achieved reaction equilibrium. Anisotropy of OPT1-DR1 complex was recorded for ∼34 days without attaining reaction equilibrium. Background was measured by unbound labeled peptide.

Anisotropy measurements were performed in black polystyrene 384-well NBS microplates (Corning) on a Tecan Infinite F200 plate reader equipped with light polarizing filters at 485 ± 20 nm for absorbance and 535 ± 25 nm for excitation, set for a 20-μs integration of 25 excitation flashes. Tecan i-Control software was used to calculate the fluorescence anisotropy defined by *A* = (*G I*_‖_ − *I*_⊥_)/(*G I*_‖_ + 2 *I*_⊥_), where *A* is the unitless anisotropy, *I*_‖_ and *I*_⊥_ are the intensity of the polarized emission parallel and perpendicular to the excitation source, respectively, and *G* is a correction factor for variance in emission detection of perpendicular *versus* parallel components. Anisotropy measurements were taken every 30 s for the first 1000 measurements and were incremented to 3, 5, 10, 15, and 20 min every 1000 measurement interval.

Observed dissociation rate constants *k*_obs_ were obtained by fitting dissociation curves to a single-phase exponential decay equation defined by *A* = *A*_0_ *e^kt^* + *A*_∞_, where *A* is unitless anisotropy, *A*_0_ is anisotropy of the peptide-MHCII complex at time zero, *k* is the observed peptide dissociation rate constant *k*_obs_, and *A*_∞_ is anisotropy of the dissociated peptide at time infinitum (reaction equilibrium). Anisotropy values were background subtracted using the free fluorescence peptide signal prior to rate modeling. Peptide dissociation rates for OPT1-DR1 complex were determined by initial velocity measurement. Rates were calculated using Prism 7.0 (GraphPad Software).

### Modeling of kinetic parameters and comparative analysis of homologous pMHCIIs

The independent kinetic parameters of DM-catalyzed peptide dissociation reactions were determined by modeling the observed peptide dissociation rates to an experimental specific kinetic model (Fig. S2),

(Eq. 1) $$k_{\text{obs}}=\frac{k_{\text{cat}}}{2{\left[p*MHC\right]}_{0}}\left({\left[DM\right]}_{0}+{\left[p*MHC\right]}_{0}+K_{m}\right)-\sqrt{{\left({\left[DM\right]}_{0}+{\left[p*MHC\right]}_{0}+K_{m}\right)}^{2}-4{\left[DM\right]}_{0}\left[p*MHC\right])}+k_{in}$$

where *k*_obs_ are the saturating dissociation rate curves obtained from anisotropy peptide dissociation assay, *k*_cat_ is the maximal dissociation rate constant (catalytic turnover) of peptide from the catalytic complex (Michaelis complex), [p*MHC]_0_ is the initial concentration of the fluorescence peptide-DR1 complex, [*DM*]_0_ is the HLA-DM concentration of the corresponding observed peptide dissociation rate constant, *K_m_* is the Michaelis constant characterizing the binding affinity of HLA-DM for the pMHCII complex, and *k*_in_ is the intrinsic peptide dissociation rate constant characterizing the intrinsic stability of the pMHCII complex. Kinetic parameters of OPT1-DR1–labeled complex were determined by Lineweaver-Burk plot analysis.

The ratio of the kinetic parameters was computed to gauge the impact of distinct peptide interactions within the pMHCII on the mechanism of DM-catalyzed peptide exchange. Comparative analysis was chosen for pMHCII homologues that varied by a distinct chemical or structural feature through a single amino acid substitution, and were bound to a stable peptide containing optimal amino acid residues in at least four of the five dominant anchor pockets, thereby eliminating ambiguity in the peptide binding registry of the pMHCII. Kinetic parameters were modeled and calculated by Prism 7.0 (GraphPad Software).

### Correlation and statistical analysis

Correlation between kinetic parameters were calculated by the Pearson *r* method to identify linear correlations and compared with the Spearman rank method to differentiate curvilinear correlations. The null hypothesis was rejected when the *p* value was <0.05. Correlations were calculated by Prism 7.0 (GraphPad Software). Statistical significance between mean values in the comparative analysis of homologous pMHCII was determined by Student's independent two-sample *t* test. The null hypothesis was rejected when the *p* value was <0.05. Student's *t* test was calculated by Stata/IC 15.0 (StataCorp). The *p* values of the analyses are as follows: *, *p* ≤ 0.05; **, *p* ≤ 0.01; ***, *p* ≤ 0.001; ****, *p* ≤ 0.0001.

## Author contributions

E. R.-V., A. P. B., Z. Z., and P. E. J. conceptualization; E. R.-V., A. P. B., Z. Z., and P. E. J. resources; E. R.-V., A. P. B., and P. E. J. data curation; E. R.-V., A. P. B., and P. E. J. formal analysis; E. R.-V., A. P. B., X. H., and P. E. J. supervision; E. R.-V. and A. P. B. validation; E. R.-V. and A. P. B. investigation; E. R.-V. visualization; E. R.-V., A. P. B., Z. Z., and P. E. J. methodology; E. R.-V. writing-original draft; E. R.-V., Z. Z., X. H., and P. E. J. writing-review and editing; A. P. B. and P. E. J. project administration; P. E. J. funding acquisition.

## Supplementary Material

Supporting Information

## Footnotes

3 The abbreviations used are:

MHCII: major histocompatibility complex class-II
HLA-DM: human leukocyte antigen-DM
pMHCII: peptide-MHC class II complex
CD4: cluster of differentiation 4
li: HLA class II histocompatability antigen γ chain (also known as invariant chain or CD74)
CLIP: class II-association invariant chain peptide
HA: hemagglutinin
HEK: human embryonic kidney.
