# Supplementary material for: HLA-DM catalytically enhances peptide dissociation by sensing peptide–MHC class II interactions throughout the peptide-binding cleft
Source: J Biol Chem. 2020 Jan 22;295(10):2959–73. doi: 10.1074/jbc.RA119.010645 (PMC7062162; doi:10.1074/jbc.RA119.010645)
Supplement: Supporting Information [file supp_295_10_2959__index.html]

HLA-DM catalytically enhances peptide dissociation by sensing peptide–MHC class II interactions throughout the peptide binding cleft — DM senses pMHCII interactions throughout the binding cleft — HLA-DM catalytically enhances peptide dissociation by sensing peptide–MHC class II interactions throughout the peptide-binding cleft — DM senses pMHCII interactions throughout the binding cleft — Supporting Information 

# HLA-DM catalytically enhances peptide dissociation by sensing peptide–MHC class II interactions throughout the peptide-binding cleft

## Supporting Information

- Supplemental Figures - Supplemental Figures 1-3
