# Supplementary material for: HLA-DM catalytically enhances peptide dissociation by sensing peptide–MHC class II interactions throughout the peptide-binding cleft
Source: J Biol Chem. 2020 Jan 22;295(10):2959–73. doi: 10.1074/jbc.RA119.010645 (PMC7062162; doi:10.1074/jbc.RA119.010645)
Supplement: Supporting Information [file supp_RA119.010645_155208_1_supp_456799_q42d3c.pdf]

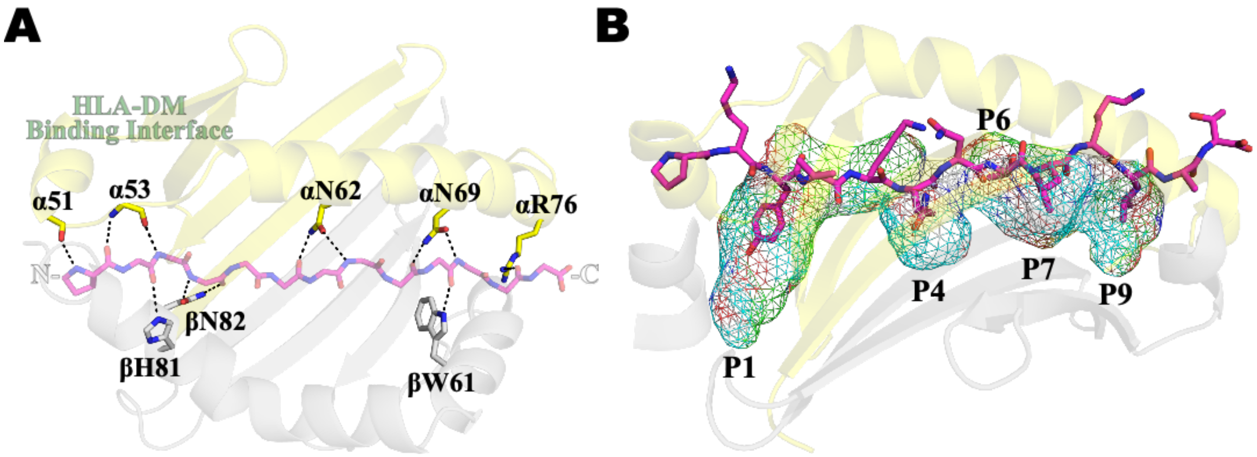

Supplemental Figure 1. Prominent structural and chemical features in the pMHCII binding cleft. MHCII molecules bind peptides within the cleft through a series of conserved hydrogen-bonds between the DR1 α/β helices and the peptide backbone and by stable interactions between peptide side-chains and anchor pockets. (*A*) A hydrogen-bond network (dashed lines), that is conserved throughout all MHCIIs, forms between the peptide main chain and α-helical DR1 α/β residues within the peptide binding cleft (top view). Hydrogen-bonds are formed by main chain DR1 α51 and α53 amino acid residues and by DR1 side-chains at all other residues. The HLA-DM binding site on DR1α chain is labeled in green. (*B*) Major anchor pockets (mesh) within the binding cleft of the HLA-DR1 complex (sliced side view). The anchor pockets vary in size and chemical properties which defines its preference for peptide residues. The pockets are N-terminally centered on the P1 amino acid position and sequentially labeled (P-1 on left and P2 on right of P1). Occupancy of the pockets defines the peptide amino acid position P1, P4, P6, P7, and P9. DR1 binding cleft is shown in ribbon diagram (DRα yellow, DRβ gray) bound with a hemagglutinin (HA) peptide shown in stick representation (main chain in *A*, sidechains in *B*). Figures were generated with PyMOL (Schrödinger, LLC) using PDB ID 1DLH.

A

$$DM + p^*MHC \overset{K_d}{\leftrightarrows} DMp^*MHC \xrightarrow[p \curvearrowright p^*]{k_{cat}} DM + pMHC$$

$$\downarrow k_{in}$$

$$pMHC$$

B

The law of mass action defines non-linear ordinary differential equations for the rate of change of reactants in reaction scheme (*A*):

$$\frac{d[p^*MHC]}{dt} = k_{off}[DMp^*MHC] - k_{on}[DM][p^*MHC] - k_{in}[p^*MHC] \quad (1)$$

$$\frac{d[DMp^*MHC]}{dt} = k_{on}[DM][p^*MHC] - (k_{off} + k_{cat})[DMp^*MHC] \quad (2)$$

$$\frac{d[pMHC]}{dt} = k_{cat}[DMp^*MHC] + k_{in}[p^*MHC] \quad (3)$$

$$\frac{d[p^*MHC]}{dt} = -\frac{d[pMHC]}{dt} \quad (4)$$

At steady-state equilibrium:

$$[DM] = [DM]_0 - [DMp^*MHC] \quad (5)$$

$$[p^*MHC] = [p^*MHC]_0 - [DMp^*MHC] \quad (6)$$

$$\frac{d[DMp^*MHC]}{dt} = 0 \quad (7)$$

Insertion of (2) into (7) after rearrangement yields

$$k_{on}[DM][p^*MHC] = (k_{off} + k_{cat})[DMp^*MHC] \quad (8)$$

Insertion of (5) and (6) into (8) after rearrangement yields

$$([DM]_0 - [DMp^*MHC])([p^*MHC]_0 - [DMp^*MHC]) = \frac{k_{off} + k_{cat}}{k_{on}}[DMp^*MHC] \quad (9)$$

Note that $(k_{off} + k_{cat})/k_{on} = K_M$, therefore (9) becomes,

$$([DM]_0 - [DMp^*MHC])([p^*MHC]_0 - [DMp^*MHC]) = K_M[DMp^*MHC] \quad (10)$$

Distributive multiplication and collection of [*DM p*MHC*] terms yields

$$[DMp^*MHC]^2 - ([DM]_0 + [p^*MHC]_0 + K_M)[DMp^*MHC] + [DM]_0[p^*MHC]_0 = 0 \quad (11)$$

This is a quadratic equation of the form $a^2x + bx + c = 0$ whose solutions are

$$[DMp^*MHC] = \frac{[DM]_0 + [p^*MHC]_0 + K_M \pm \sqrt{([DM]_0 + [p^*MHC]_0 + K_M)^2 - 4[DM]_0[p^*MHC]_0}}{2} \quad (12)$$

The reaction scheme (*A*) is recorded by a condensed fluorescence anisotropy reaction scheme

$$DM + p^*MHC \xrightarrow[p \curvearrowright p^*]{k_{obs}} DM + pMHC$$

whereby $[DM] \approx [DM]_0$ = constant gives rise to the rate of change of [*p*MHC*]

$$\frac{d[p^*MHC]}{dt} = -k_{obs}[p^*MHC] \quad (13)$$

Inserting (3) and (13) into (4) results in

$$k_{obs}[p^*MHC] = k_{cat}[DMp^*MHC] + k_{in}[p^*MHC] \quad (14)$$

Utilizing the steady-state approximation and solving for k_{obs} yields

$$k_{obs} = \frac{k_{cat}}{[p^*MHC]_0}[DMp^*MHC] + k_{in} \quad (15)$$

Inserting (12) into (15) and choosing the saturable root gives the equation in panel (*C*)

C

$$k_{obs} = \frac{k_{cat}}{[p^*MHC]_0}\left(\frac{[DM]_0 + [p^*MHC]_0 + K_M - \sqrt{([DM]_0 + [p^*MHC]_0 + K_M)^2 - 4[DM]_0[p^*MHC]_0}}{2}\right) + k_{in}$$

Supplemental Figure 2. A Michaelis-Menten kinetic model resolves the kinetic parameters of HLA-DM catalyzed peptide exchange reactions. We developed a fluorescence anisotropy assay that records the real-time dissociation of peptides from an MHCII molecule in the presence of titrated amounts of HLA-DM. Anisotropic dissociation curves were fit to a single phase exponential decay formula to ascertain the observed peptide dissociation rate (k_{obs}) at each HLA-DM concentration. (*A*) The observed DM-catalyzed peptide dissociation rates are consistent with a Michaelis-Menten kinetic reaction scheme, whereby the reversible rapid steady-state binding of DM to a fluorescence-labeled peptide–MHCII complex assembles the DM–pMHCII catalytic complex (Michaelis complex), by a rate given by the equilibrium dissociation constant K_d (K_M in our derivation), and dissociates from the pMHCII by the rate-limiting, experimentally irreversible (excess unlabeled competitor peptide) dissociation of the peptide from the catalytic complex, as characterized by the catalytic turnover constant k_{cat}. The reaction scheme includes the peptide dissociation in the absence of HLA-DM whose rate is given by the intrinsic dissociation rate constant k_{in}. (*B*) An experimental specific equation was derived that relates the observed peptide dissociation rates to DM-binding affinity, catalytic turnover, and the intrinsic stability of the pMHCII. (*C*) The equation models the Michaelis-Menten reaction scheme and relates the observed peptide dissociation rate k_{obs} with the independent kinetic parameters of the HLA-DM catalyzed reaction: catalytic turnover number k_{cat} as a measure of the stability of the DM–pMHCII catalytic complex; the Michaelis constant K_M as a measure of DM binding affinity to a pMHCII complex; and the intrinsic peptide dissociation rate k_{in} as a gauge of the intrinsic stability of the pMHCII complex. An asterisk (*) denotes the peptide fluorescence label.

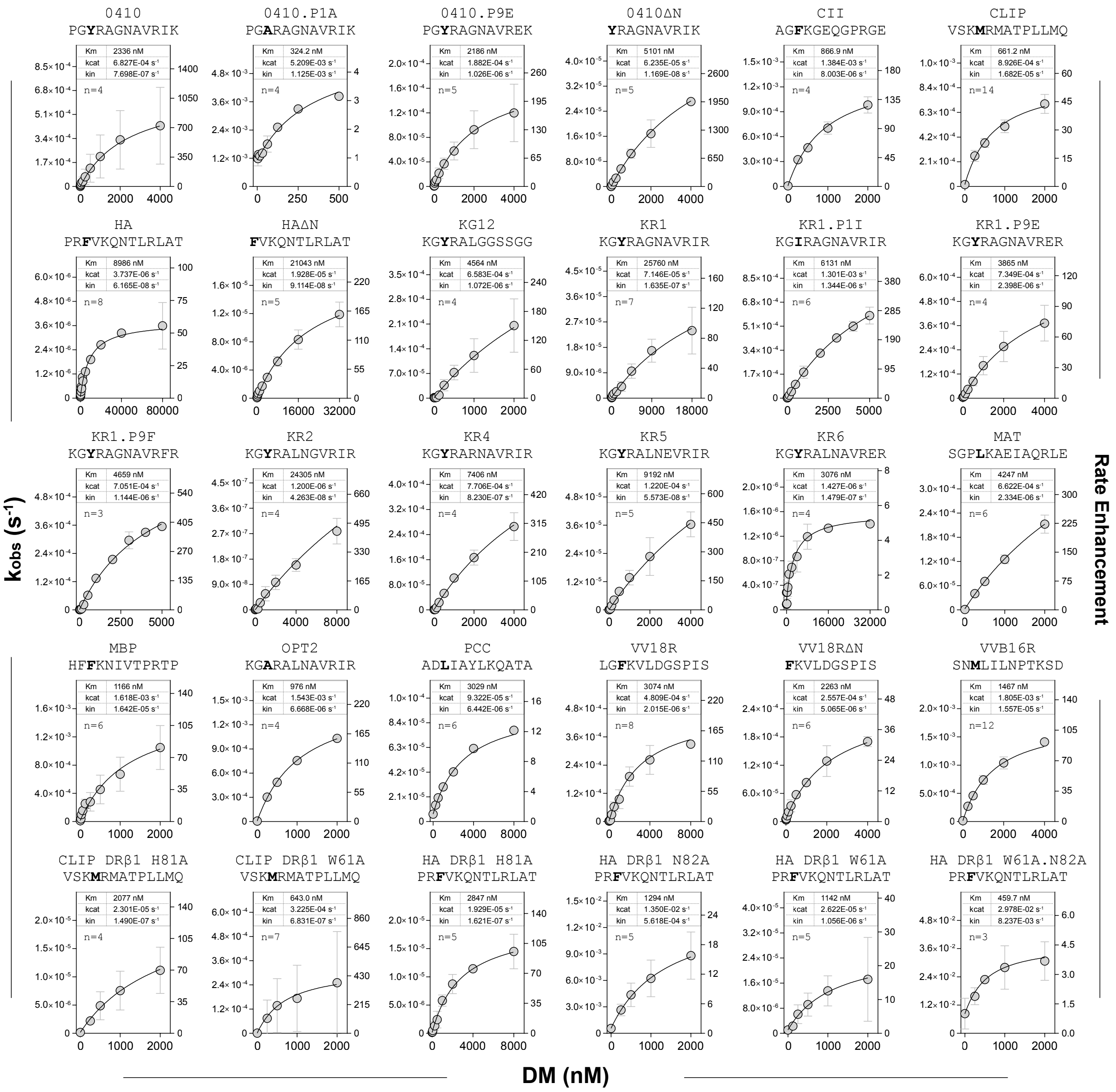

Supplemental Figure 3. Saturating rate curves of HLA-DM catalyzed peptide dissociation of HLA-DR1 wild-type and mutant complexes. Anisotropic peptide dissociation curves of fluorescence-labeled peptides bound to HLA-DR1 were fit to a single phase exponential decay equation to compute the observed peptide dissociation rate k_{obs} at each concentration of HLA-DM. Saturating curves of the observed peptide dissociation rates were fit to an experimental specific Michaelis-Menten kinetic model (Supplemental Fig. 2) that resolved the kinetic parameters of the DM-catalyzed peptide exchange reaction for each DR1–peptide complex. Left axis denotes the observed peptide dissociation rates (k_{obs}). Right axis indicates the fold rate enhancement over the intrinsic peptide dissociation rate (k_{in}). Graph titles identifies the pMHCII complex and peptide sequence. The kinetic values and number of independent experiments (n) performed are given in the inset. Mean ±SD values are shown as solid circles from data of at least three independent experiments.
